# PaxDb v6.0: reprocessed, LLM-selected, curated protein abundance data across organisms

**DOI:** 10.1093/nar/gkaf1066

**Published:** 2025-11-03

**Authors:** Qingyao Huang, Damian Szklarczyk, John Oehninger, Christian von Mering

**Affiliations:** Swiss Institute of Bioinformatics, Winterthurerstrasse 190, 8057 Zurich, Switzerland; Department of Molecular Life Sciences, University of Zurich, 8057 Zurich, Switzerland; Swiss Institute of Bioinformatics, Winterthurerstrasse 190, 8057 Zurich, Switzerland; Department of Molecular Life Sciences, University of Zurich, 8057 Zurich, Switzerland; Department of Molecular Life Sciences, University of Zurich, 8057 Zurich, Switzerland; Swiss Institute of Bioinformatics, Winterthurerstrasse 190, 8057 Zurich, Switzerland; Department of Molecular Life Sciences, University of Zurich, 8057 Zurich, Switzerland

## Abstract

Proteomics captures the biological and functional state of cells, condensing complex molecular information into quantitative measurements, yet the reuse of public mass spectrometry (MS) data is impeded by heterogeneous processing and incomplete metadata, limiting its potential to generate new biological insights. These issues restrict reproducibility and cross-study integration, underscoring the need for standardized, high-coverage reference resources. PaxDb addresses this by providing a protein abundance reference at organism- and tissue-level, for the healthy, wild-type state. The v6.0 release integrates 1639 datasets from 392 species, nearly doubling coverage since v5.0, with expanded representation across all kingdoms of life. A new end-to-end MS data processing pipeline enables consistent re-analysis from raw files using the FragPipe framework, integrating standardized metadata, orthology mappings, and protein–protein interaction-based quality scoring. To our knowledge, this is the first large-scale, unbiased, automated reprocessing of public MS data, including links to metadata. We further developed large-language model ensemble classifiers to semi-automate the curator-selection of relevant ProteomeXchange (PX) projects, as well as a user-facing tool for peptide-level abundance calculation, dataset scoring, and direct comparison with PaxDb reference data. The updated database is available at https://www.pax-db.org.

## Graphical Abstract

**Graphical Abstract:**
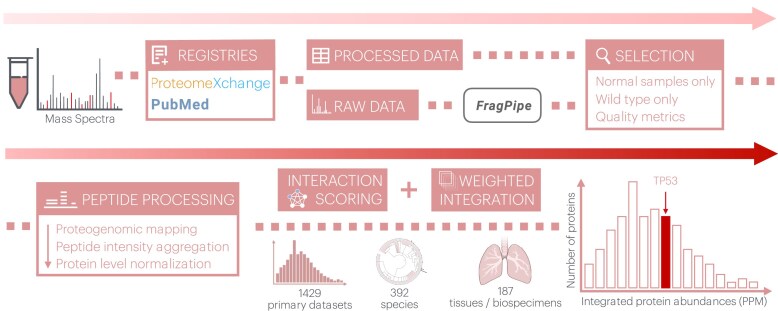

## Introduction

Proteomics research holds a distinct position among the omics disciplines. While genomics concerns the stable blueprint across generations, transcriptomics provides a snapshot of the transient cellular states, and metabolomics reports on the status of synthesized small molecules, proteomics directly measures the functional machinery operating within and outside cells. Proteins are the principal executors of biological functions, and their turnover has been shown to be partially decoupled from transcript levels, adding an additional layer of expression regulation [1–3]. The proteome exhibits a far greater dynamic range (∼10^6^, and up to 10^10^ in some samples) compared to the transcriptome (∼10^3^) [4]. In conditions where normal cellular functions are disrupted, directly measuring responses at the proteome level is essential for understanding mechanisms [5]. In particular, protein biomarker discovery is critical for diagnosis and disease assessment, and recent technological advances have enabled analysis of previously challenging sample types, such as body fluids [6, 7]. Another emerging direction is the study of immunopeptide diversity to better understand autoimmune and autoinflammatory diseases, cancer, and infectious diseases [8]. Characterizing context-specific protein–protein interactions (PPIs) is also an active area of research, linking molecular functions to their clinical context. Techniques such as affinity purification and, more recently, proximity labeling have enabled accurate and efficient measurements of dynamic, condition-dependent PPIs [9]. Studying both differential expression and changes in PPIs requires a baseline of undisturbed, healthy-state proteome measurements, ideally including stoichiometry. PaxDb is a resource that curates such information, focusing on protein abundance measurements in tissue-specific and whole-organism proteomes under normal physiological conditions. It aggregates data from published proteomics experiments and provides both individual datasets and integrated organism-wide and tissue-level summaries, expressed in relative quantification units [i.e., number of molecules of a given protein of interest, relative to all protein molecules in the cell, in parts per million (ppm)]. Several other resources offer related data. PeptideAtlas [10] compiles peptides identified from mass spectrometry (MS)-based proteomics experiments and regularly releases builds for human, mouse, yeast, and other organisms—summarizing datasets, experiments, MS runs, spectra, peptide–spectrum matches (PSMs), distinct peptides, and canonical core proteins peptideatlas.org. ProteomicsDB [11] integrates MS-based proteomics with RNA-seq expression profiles, drug–target interactions, cell-line viability assays, and protein turnover measurements; in addition to its coverage of four model organisms (human, mouse, Arabidopsis, and rice), it recently incorporated over 300 bacterial species from a large-scale bacterial proteomics project [12]. QuantMS [13] provides both a cloud-based reanalysis pipeline and a data repository comprising 13 132 human proteome samples. Single-cell Proteomic DataBase (SPDB) [14] focuses exclusively on single-cell proteomics data. Other resources include large-scale single studies, such as the Proteomes of Life [15] and the Proteomic Diversity in Bacteria [12]. When available, these data sources are incorporated in PaxDb during its periodic updates.

In contrast to these resources, PaxDb sources its data from a broad range of independent publications selected according to a clearly defined scope, covering all species rather than focusing solely on large-scale or collaborative projects. Its specific aim is the quantification of the whole proteome—at the organism or tissue level—under healthy, normal conditions.

The datasets included in PaxDb typically fall into two categories: (i) studies quantifying a large fraction of the entire proteome in simple organisms or the whole proteome in tissues of higher organisms; and (ii) studies quantifying proteomes under different conditions that include healthy control samples for differential expression analysis. By design, PaxDb excludes data sets from cross-linking, affinity purification, or biochemical fractionation studies that quantify only a subset of the proteome, environmental samples containing multiple species, studies comparing disease and treatment conditions, mutational or viral studies, subcellular or organelle proteomes, datasets focused exclusively on post-translational modifications, archaeological samples, synthetic material or chemical studies, as well as protocol development work. PaxDb presents its selected datasets with standardized, ontology-based metadata to support interoperability and ease of downstream use. Gene orthologies are drawn from eggNOG [16], enabling cross-organism comparisons, while tissues and biospecimens are encoded in the following ontologies: Uber-anatomy ontology (UBERON) [17], Plant Ontology (PO) [18], Cell Line Ontology (CLO) [19], Cell Ontology (CL) [20], and The BRENDA Tissue Ontology (BTO) [21]. Full data provenance is maintained through links to the source publication’s PubMed IDs and the ProteomeXchange (PX) accession IDs, facilitating data rediscovery and reanalysis.

The PX consortium [22] underpins much of PaxDb’s data provenance strategy. PX provides a standardized data submission system that assigns a unique accession ID to each project, publishes its metadata, and links to the raw and processed files hosted in member repositories. Since most proteomics publication venues now require deposition of supporting data for reproducibility—and because proteomics datasets generally contain no sensitive information—the volume of publicly available data continues to grow rapidly. The PX community has incorporated the FAIR principles (findable, accessible, interoperable, reusable) [23, 24] into its data schemas and interfaces, promoting systematic sharing and reuse of proteomics data. However, beyond availability, effective reuse requires standardized annotation of data files. The Sample and Data Relationship Format (SDRF) [25] was developed to link experimental design and sample information directly to the corresponding data files. Tools such as lessDRF [26] aim to encourage adoption of this standard, and the FragPipe software framework now generates SDRF files automatically as part of its workflow. Despite these developments, most submitted projects still lack SDRF annotation.

In 2024 alone, over 12 000 projects were submitted to PX, underscoring both the scale of available data and the challenges of systematic reuse. This rapid growth reflects a broader acceleration in proteomics, driven by advances in sample preparation, transformative instrumentation, and innovative data acquisition strategies. These developments now enable measurement at the single-cell level, detection of unexpected post-translational modifications, and preservation of spatial information [27]. In parallel, improvements in data processing—such as DIA-NN [28], which provides accurate and computationally efficient quantification of complex spectra from data-independent acquisition (DIA) experiments on modern mass analyzers—have increased the depth and reliability of analyses. Automated software frameworks such as FragPipe [29] further streamline reanalysis, while tools such as FragPipeAnalyst [30] and Maxquant Perseus [31] offer comprehensive analysis environments for individual experiments, facilitating data exploration and reuse.

Against the backdrop of this rapid technological growth, PaxDb has expanded its data content primarily through manual literature curation and inclusion of quantification data as processed by the original authors. However, proteome-wide quantification results are not available for many projects, and updated genome sequences enable more accurate peptide identification and quantification when reprocessing the original spectral data [32]. To address these gaps, we implemented an end-to-end data processing pipeline that operates directly from raw files, incorporating file name grouping, metadata parsing, parallelized processing, and quality control, using the FragPipe framework as its core engine.

At the same time, the manual project screening process cannot keep pace with the accelerating growth of proteomics data. Leveraging recent advances in large-language models (LLMs) and open infrastructure, we developed LLM-based ensemble classifiers to preselect projects relevant to PaxDb’s scope, increasing coverage while reducing curation effort. Finally, we introduce a web interface that enables users to run their own MS-based proteomics data through the PaxDb processing pipeline, apply STRING-based [33] PPI quality scoring, and compare results with PaxDb reference datasets of the same species when available.

Together, these developments transform PaxDb from a manually curated reference into a semi-automated, extensible platform for large-scale, standardized, and reusable protein abundance data.

## Materials and methods

### Large-language model ensemble classifier

An ensemble classifier was trained to help in the data curation process, by suggesting suitable datasets for processing and inclusion.

#### Labeled data

Only publications linked to PX projects are used for data labeling to ensure that the publications cover the proteomics field and match candidate texts to be classified for curation later. A labeled text set of size 1139 was curated, consisting of the title and abstract of publications referred in the “Publication” field in a PX Dataset’s metadata. Two hundred one positive labels were derived from the previously included projects, and the rest of the 938 publications were chosen randomly from the publication pool, in which 200 were evaluated as positive according to the scope described in Introduction and 738 were labeled negative for meeting one or more exclusion criteria. The positive-to-negative ratio in the pool is estimated as 200/738.

Using the labeled data, we employed three groups of methods to classify the input texts: topic modeling (TM), embedding-based classification, and OpenAI Chat Completions prompts.

#### Topic modeling with BERTopic

TM was performed using BERTopic [34] with the Python implementation by MaartenGr/BERTopic. First, the embeddings were pre-computed from the original texts with existing embedding models. Then the dimensionality of the embeddings was reduced with UMAP. HDBSCAN was used to cluster the embeddings. Finally, a class-based TF-IDF was used to generate keywords representing the topic. The topic clusters were sorted based on the proportion of positive labels within the cluster and included until the proportion dropped to 0.5 or the outlier group (topic-1) was reached.

Three embedding models, “Alibaba-NLP/gte-Qwen1.5,” “thenlper/gte-small,” and “thenlper/gte-large” were tested. English stop words and “proteomics”-related stop words were tested for preprocessing. For UMAP, the number of neighbors was tested between 3 and 10, and the number of components between 5 and 15. For HDBSCAN, the minimal number of class sizes was tested between 10 and 30.

These parameters were trained with the Optuna† hyperparameter optimization framework [35] with the Area Under the Curve (AUC) of positive rate versus topic inclusion rank plot as optimizing objective and with 250 trials.

The performance (recall, precision) was evaluated with the five-fold cross-validation on the labeled dataset and the median was used for evaluation. The summary was ranked by F1 to choose the top models for ensemble voting.

#### Embedding classification

We developed a classification framework that leverages transformer-encoder-based models (BERT). Titles, abstracts, and keywords were embedded using multiple pretrained models, namely “allenai/longformer-base-4096” [36], “answerdotai/ModernBERT-large” [37] and “Simonlee711/Clinical_ModernBERT” [38]. From those, two types of vector representations were extracted: the [CLS] token embedding and the mean-pooled embedding across all tokens. In a separate attempt, each of the pre-trained BERT models was fine-tuned end-to-end as a binary classifier using supervised learning. Dimensionality reduction via PCA was applied to the extracted embeddings to reduce overfitting, and the resulting representations were used to train a suite of classical machine learning classifiers, including logistic regression, support vector machines, random forest, and XGBoost. All models underwent hyperparameter optimization using Optuna [35] with Bayesian search, targeting both F1 and F0.5 metrics in an attempt to increase precision over recall. Finally, majority voting was conducted with an ensemble of the top-performing models in order to ensure an overall robust prediction on the new data.

#### OpenAI Chat Completions API

OpenAI Chat Completions API with GPT-3.5 and GPT-4o models was tested. The texts used were title and abstract of each publication. The following parameters were used: temperature = 1.0, top_p = 0.75, and max_token = 5. With max_token set at 5, the API answer was limited to yes or no and was compared with the label to evaluate the performance. Multiple versions of prompts were tested with the labeled dataset. The best prompt that generated the performance in Table 1 is in Supplementary Material 1.

**Table 1.** Performance metrics for models

Method	Instances	Recall	Precision	F1	Effective* precision
	R10_6_5_3	0.609	0.786	0.691	
TM	S10_6_5_2	0.804	0.62	0.682	66/103
	R11_8_5_1	0.77	0.6	0.691	(64.1%)
	FT008	0.767	0.747	0.757	
EC	FT010	0.562	0.854	0.678	69/96
	CM109	0.795	0.58	0.671	(71.8%)
OpenAI	GPT-3.5	0.798	0.515	0.626	
	GPT-4o	0.612	0.711	0.658	
Keyword		0.672	0.512	0.581	
Random	50–50	0.5	0.352	0.413	

* By manual evaluation on projects at CP4.

### End-to-end MS-based proteomics data processing workflow

From the shortlisted projects suggested by the classifier models, the metadata were downloaded from PX, where the raw file names and respective URLs to download the raw data were extracted. The file names were parsed and grouped as follows:

#### File name grouping

In order to merge technical and biological replicates into the same group and separate data files by experimental conditions/setups, we had to use the information from the file names directly. For each data file name, common word separators were converted to underscores, numbers were removed, camelCase or PascalCase strings were converted into snake_case, and finally, the underscores were converted to spaces. Subsequently, the string was tokenized with the SpaCy NLP model “en_core_web_md.” Additionally, the strings (“wt,” “ctrl,” “ko,” and “organoid”) as well as a parsed list of cell line names from Cell Line Ontology [20] were added to the token list. Tokens with less than three letters were excluded. Then, the file names were grouped based on the combination of tokens each file name contains.

For the projects with successful grouping outcomes, where technical and biological replicates could be distinguished from different conditions, the taxonomy IDs of the metadata-stated species were mapped to species in STRING database [33]. From there, the FASTA files for the protein sequences as well as the protein–protein interaction files were downloaded. The raw files were downloaded from the respective repositories. From the FASTA files, decoy databases were generated using the DecoyDatabase component from the openMS framework [39] with cRAP (The common Repository of Adventitious Proteins) list of contaminants downloaded from the Global Proteome Machine [40]. Then the input files (.manifest and .workflow files) for the FragPipe framework [29] were created and processed as follows:

#### MS Data processing

To process the MS-derived raw data, the FragPipe framework was used. Fragpipe v22.0 was opened in the graphical user interface to export the workflows and manifest templates for running in headless mode. The DIA-NN version 1.8.1.8 with EasyPQP version 0.1.52 was used for DIA processing. The Fragpipe workflow was set to allocate 128 GB RAM and to run 42 threads in parallel.

For label-free experiments performed in data-dependent acquisition (DDA) mode, the “lfq-mbr” workflow template performs closed search, label-free quantification with match-between-runs with IonQuant. Parameters for multiple enzymes and their combinations, e.g. trypsin, gluC, lysC, and lysN, were extracted.

To process experiments performed in DIA mode, a DIA workflow was built from “DIA_SpecLib_Quant” workflow template, which performs a direct search on DIA data with MSFragger-DIA, spectral library generation with EasyPQP, and quantification with DIA-NN. Additionally, the .raw files were converted to .mzML format in a Podman instance running msconvert from the container image “chambm/pwiz-skyline-i-agree-to-the-vendor-licenses” with a Windows executable [41]. The “report.tsv” file in the “diann-output/” folder was converted to “combined_protein.tsv” file to make it compatible with the downstream process.

Labeled experiments were processed with customized workflows; e.g. a dimethyl labeling workflow was generated with the “SILAC3” template, which performs a closed search with MSFragger and labeled quantification with IonQuant. The variable modifications and MS1 light/heavy labels were set following the developer guidelines. The output files were also converted with regard to the label-specific columns into the standard format for downstream processing.

SDRF files were created and made available together with the data for reproducibility.

If the data processing finished successfully, “combined_peptide.tsv” and “combined_protein.tsv” files were generated. The scope of the project was then manually checked, and human-readable dataset labels and tissue ontology terms were assigned as follows:

#### Scope, label, and tissue curation

The manual curation was performed only if the previous processing steps were successful. First, the project scope was reviewed according to the criteria, given the title and abstract of the PX-project-affiliated article as well as the project title and description. The rejection reason was recorded for further analysis and future training.

The group label was assigned according to the file name and the project description. The tissue label was assigned based on the PRIDE “Organism part” of the project, if available, or derived from the project description.

In the group label assignment step, any grouping errors were corrected, e.g. if a suitable keyword was not identified for correctly separating two distinct groups, then a “-curated” .manifest file was manually created with corrected sample groups, and the files were reprocessed.

Finally, the “combined_peptide.tsv” and “combined_protein.tsv” files were used to compute the abundance with the NSAF (normalized spectral abundance factor)-based pipeline [42, 43] or iBAQ pipeline (as described in [32]), respectively. The dataset quality scores were computed for peptide- or protein-derived datasets using PPI information from STRING (as described in [44]). By default, the peptide-derived dataset is used unless both the score and coverage are higher in the protein-derived dataset data.

### Version update streamlining

The 6.0 version update is aligned with STRING Database [33] version 12, with respect to the included species and their taxonomy identifiers, internal protein identifiers and namespaces, protein sequences, and the PPI networks used for quality control. Changes in species taxonomy assignments between PaxDb 5.0 and 6.0 were resolved using the NCBI taxonomy tree. If the original data in a v5.0 dataset consists of peptide abundances, the NSAF method is rerun with the updated species FASTA file, to generate new protein abundance data. For datasets where the original data consists of protein identifiers, the abundances are lifted over to the updated v6.0 proteomes using sequence homology via the reciprocal best hit method. With these mappings, the datasets between major versions are lifted over. During the liftover from v5.0, 26 species have changed the taxonomy ID, but the proteomes could be remapped entirely or almost entirely. The datasets lifted over from v5.0 were highly consistent in terms of coverage (Pearson’s *r* at 0.997) and quality score (0.949). Three species (*Bubalus bubalis, Lactococcus lactis*, and *Clostridium bolteae*) and eight of their associated datasets have been archived, as these species have been removed from the current version for archival.

With continuous integration of new datasets, minor updates are announced between major updates. After the data processing pipeline is run, the datasets are added to the existing PaxDb data. Subsequently, the species/tissue integrated datasets are updated. With the finalized protein list, the PostgreSQL database for species and protein information is created and used for protein search. As well, the Neo4j graph database storing protein-ortholog information is built. Finally, the static files (downloads, cross-reference files, taxonomy files, logo, image, and sources) as well as the website are rebuilt. Both major and minor updates trigger reintegration on the species or tissue level and the subsequent data-dependent databases, API, and website updates (Supplementary Fig. S1).

## Results

### Data update

**Figure 1.**
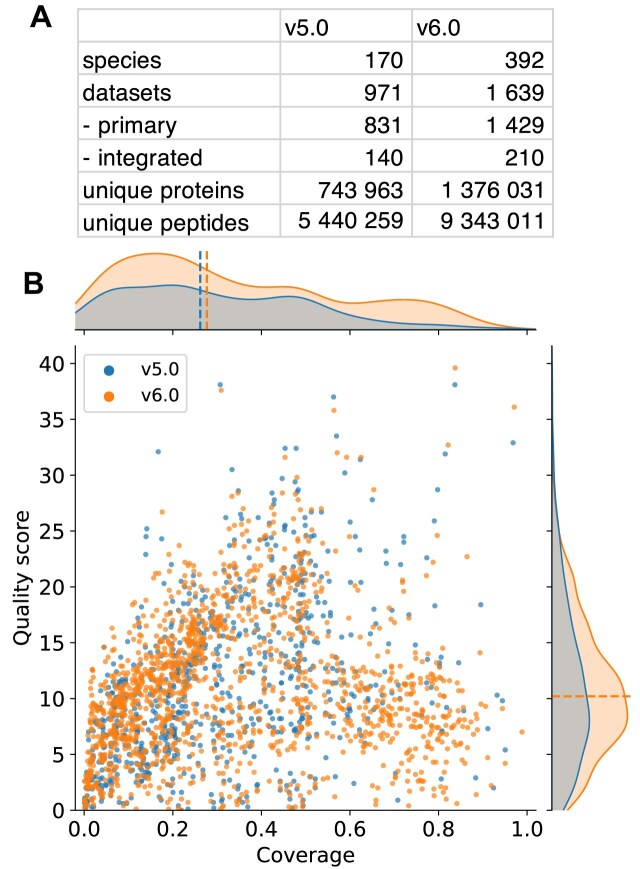
Data update statistics. (**A**) Count statistics on the datasets, species, proteins, and peptides. (**B**) The dataset quality score and proteome coverage among primary datasets in v6.0 (1429) and v5.0 (821).

Corresponding to STRING v12 and eggNOG v6 for namespaces and orthologs [16], the updated PaxDb database v6.0 contains 1639 datasets (971 in v5.0) for 392 species (170 in v5.0): 1005 from Eukarya, 382 from Bacteria, and 39 from Archaea. One thousand four hundred twenty-nine of these are primary datasets with reference to the original experiments and 210 are aggregated (“integrated”) at the species or tissue level. PaxDb currently covers 1 376 031 unique proteins (743 963 in v5.0) and 9 343 011 unique peptides (5 440 259 in v5.0) (Fig. 1A). In v5.0 and before, the newly added data had been manually selected, typically corresponding to large-scale, high-impact flagship studies. In v6.0, we have introduced a semi-automatic project selection and inclusion pipeline, which added 359 new datasets to the database. Additionally, we have included data from a large bacterial study (MSV000096603) [12], comprising 219 new datasets. The tissue-specific data statistics can be found in Supplementary Material 2. Last but not least, we incorporated three recent builds from PeptideAtlas: *Labeo rohita* (Build 2020-07) [45], *Oryza sativa* (Build 2025-03), and *Canis lupus* (Build 2024-01).

The median proteome coverage shifted up to 0.28 from 0.26, while the dataset quality score stayed the same with a median at 10.2 in v5.0 (Fig. 1B). The coverage and quality scores show a significant positive correlation despite different strengths among the three kingdoms (Supplementary Fig. S2A). With the additional data, the integrated datasets show an increase in coverage (median coverage from 0.42 to 0.46) while staying at similar quality scores (median 15.0 to 14.9) when compared to the overlapping categories in v5.0 (Supplementary Fig. S2B). We also observe a shift toward more recently developed and more advanced MS instrumentation, as compared to the previous version. Orbitrap Exploris (29.6%, launched in 2019) superseded Q Exactive (24.0%, launched in 2012) as the most used instrument, with Q Exactive HF (14.8%) and Orbitrap Fusion (7.8%) closely following (Supplementary Fig. S2C).

### End-to-end MS data processing workflow

**Figure 2.**
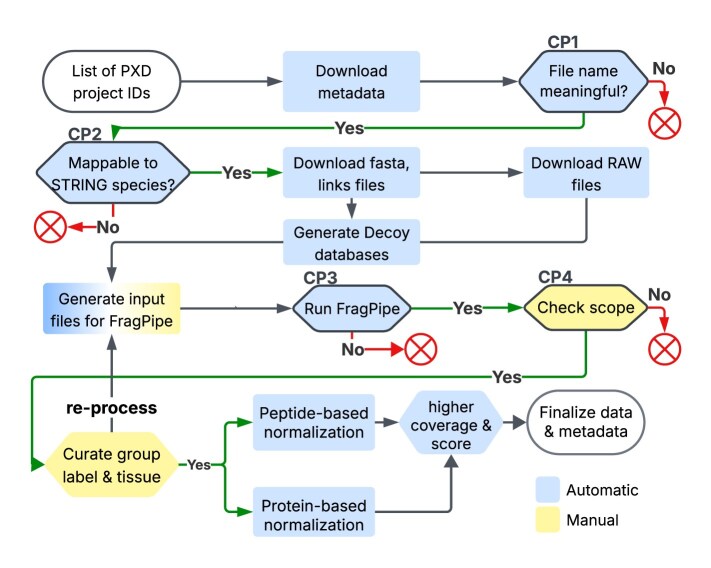
Data processing workflow. Four CPs indicate steps where projects may drop out.

With this version, we have introduced an end-to-end MS data processing workflow (Fig. 2). The workflow begins with a list of project IDs from PX. The file names extracted from the downloaded metadata are parsed and analyzed, with the aim of grouping the samples into experiment/condition replicate groups. The species information from the parseable projects is mapped to the STRING species, and for successfully mapped species, the corresponding protein sequences from STRING serve as references. Using this workflow, we have included data from 146 projects from PX, corresponding to the 359 new datasets in this update. There are four checkpoints (CPs) where projects might drop out of the workflow. CP1 checks the file name parsing. A typical project may consist of dozens of raw data files. The parser step relies on file names being readable and meaningful, namely, containing valid tokens described in Section File name grouping. The number of eligible projects at the beginning of the processing was 3202. At CP1, 541 remained where file names could give a hint of the sample information. CP2 checks whether the species is included in STRING protein interaction database, as the quality control of protein abundance data depends on the functional association scores. In this update, after CP2, 475 projects remained. CP3 checks whether the peptide and protein summary files are correctly generated after the processing has finished. 267 projects passed CP3. CP4 is based on manual evaluation. The project title and description, as well as the associated publication’s title and abstract, are taken together to decide whether the project fits the PaxDb scope (see the “Introduction” section). If it is suitable, the automatic groupings and group labels are checked to ensure the processing parameters were correctly set, and the tissue ontology IDs are assigned for applicable datasets. In case of unrecognized experimental conditions like DIA or alternative digestion enzymes, or incorrectly recognized groups, reprocessing is performed with adjusted parameters. In this update, 64 projects have been reprocessed and in total 146 projects have passed the scope check and sample labeling to be included in PaxDb.

During the project analysis, we have encountered overlaps with previously curated PaxDb datasets, for which the original quantification results uploaded by the authors had been used. This allowed a comparison between our processing output and the author’s results. (Supplementary Fig. S3A) For all eight datasets, the quantified proteome coverage is now higher, and six of them also have a higher quality score.

We also summarized the processing requirements, in terms of run-time and system resource footprint, of a typical experiment. (Supplementary Fig. S3C and E)

### LLM-based project selection

With the established end-to-end workflow, pre-selecting the projects by their scope becomes the critical step to increase the chance of data inclusion and to avoid unnecessary processing.

We tested different approaches, using a manually assembled gold-standard set of 1139 publications, labeled according to whether or not they fall within PaxDB’s scope (401 of these do, and hence receive a positive label). Then, we ran the winning methods on the unlabeled set of 17 503 PX-project-associated publications collected until June 2024. From the LLM-based classifiers built for the project selection, we have observed large performance differences from the tested method groups.

First, like in previous versions of PaxDB where dataset selection had been done manually, we used a simple keyword search as a baseline for the performance evaluation. The appearance of either “quant” or “abundance” in the title and abstract gave the highest F1 score (0.581) with 0.672 recall and 0.512 precision. This is an improvement over a random guess on the labeled set, which would result in a precision of 0.352 (401/1139). With the keyword filter, 6417 would be classified as initially suitable for PaxDb from the unlabeled texts.

Then, in the TM method group, we used the BERTopic analysis framework, which clusters texts into topic classes. We ranked the classes by the ratio of positive over negative labels as a fitness measure. Then, with an AUC of the number of positive labels covered by the top topic classes until the set threshold, the embedding, dimensionality reduction, and clustering parameters were optimized. The F1-ranked top 3 models had a recall between 0.609 and 0.804 and a precision between 0.6 and 0.786. They were used to derive the consensus choice on the unlabeled texts. Four thousand three hundred forty-two projects were voted positive by at least two models and 1368 projects were voted positive by all three models (Supplementary Fig. S4A).

Next, in the embedding classification (EC) method group, we used contextual embedding of the texts as input to train a classifier. The top two models ranked by F1 were fine-tuned LLMs. The third best was a contextual embedding-based model. They had a recall between 0.562 and 0.795 and a precision between 0.58 and 0.854. Two thousand three hundred sixty-three projects were voted positive by at least two models and 1285 projects were voted positive by all three models (Supplementary Fig. S4B).

Lastly, we tested the OpenAI Chat Completions API. With the same prompt, the GPT-4o model was more conservative in approving a text as positive, resulting in higher precision and lower recall than GPT-3.5. Overall, its F1 score was still higher than GPT-3.5 model and would be the preferred choice, but we did not run the API for the full set of unlabeled texts for processing, as the two non-commercial classifiers had better performance.

The consensus projections from the Top3 EC models and the Top3 TM models were compared with the keyword filter outcome; the overlap of the three dropped to 125 projects only (Supplementary Fig. S4C), which indicates that the methods pick up different aspects of the text input.

We then performed a final manual evaluation. Because the keyword filter output covers >1/3 of the projects, we ran the end-to-end workflow only on the Top3 consensus projects from the TM methods (1368) and the EC methods (1285). Then, we evaluated the project scope at the CP4 step from Fig. 2. From the 103 projects derived from the TM method, 66 were positive, resulting in 64.1% effective precision. From the 96 projects derived from EC method, 69 were positive, resulting in 71.8% effective precision. All the methods’ performances are summarized in Table 1.

### User-uploaded data processing

**Figure 3.**
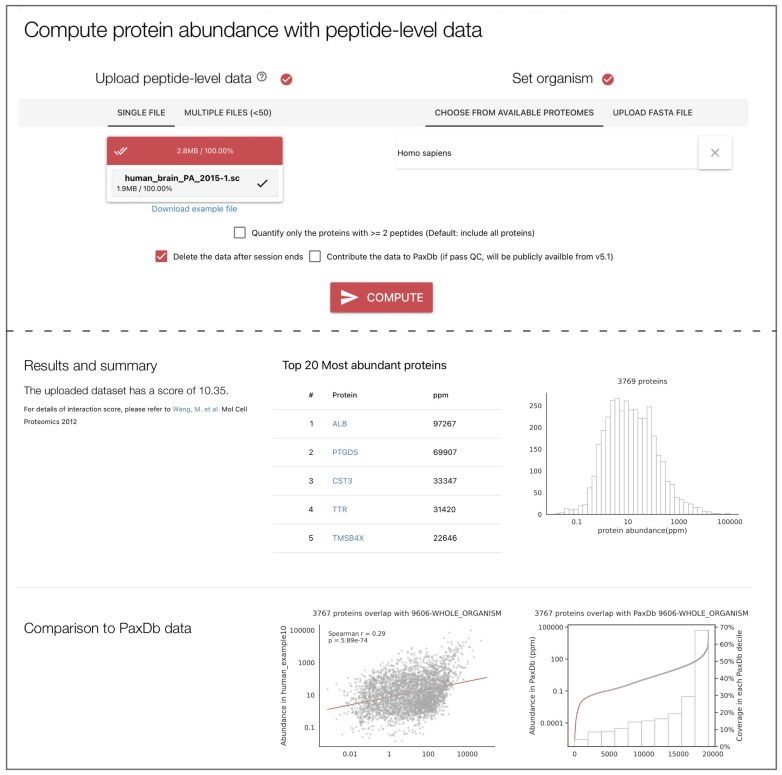
The user peptide data upload web interface allows users to compute protein abundances and compare with PaxDb reference data, if available.

We have developed an online tool to process user-uploaded data for abundance computations. The data input format is currently restricted to peptide counts or intensities. Uploads of multiple files (up to 50) are allowed. In addition to the peptide data, a reference proteome is required, which can be obtained by selecting the taxonomy ID from the available species in STRING or by uploading a custom protein fasta file. Users can choose to contribute the data to PaxDb by providing the dataset information and contact information for reference. Otherwise, by default the dataset is deleted after being processed. By default, all peptides contributing to proteins are taken into account, but users can also choose to restrict the output to proteins confirmed by at least two peptides. With these inputs, the server runs the NSAF method as used in the PaxDb internal pipeline. If the taxonomy ID was selected from the STRING species, a protein-interaction quality score is computed with the method used for PaxDb dataset score [44]. Next to it, a table of top 20 most abundant proteins is shown, as well as an abundance histogram of the computed data. If PaxDb already contains data of the same species, a scatterplot can be created to show the dataset’s correlation with PaxDb reference data on the overlapped proteins. A second quality control plot is made with ranked dot plots showing the dynamic range in gray superimposed on the PaxDb reference abundance in red and a bar plot showing the percentage of computed dataset covering the PaxDb abundance decile range. In the example below, of the top 10% abundant PaxDb proteins, 67% were present in the user data, but of the bottom 10% abundant PaxDb proteins, only 4% were present (Fig. 3).

### Use cases

Finally, we present two use cases demonstrating application of PaxDb data to uncover biological patterns. First, we assess the “housekeepingness” of genes on the protein level in terms of their constitutive expression. Second, we performed a comparative analysis on orthologous genes’ abundance conservation.

#### Evaluation of housekeeping genes on the protein level

**Figure 4.**
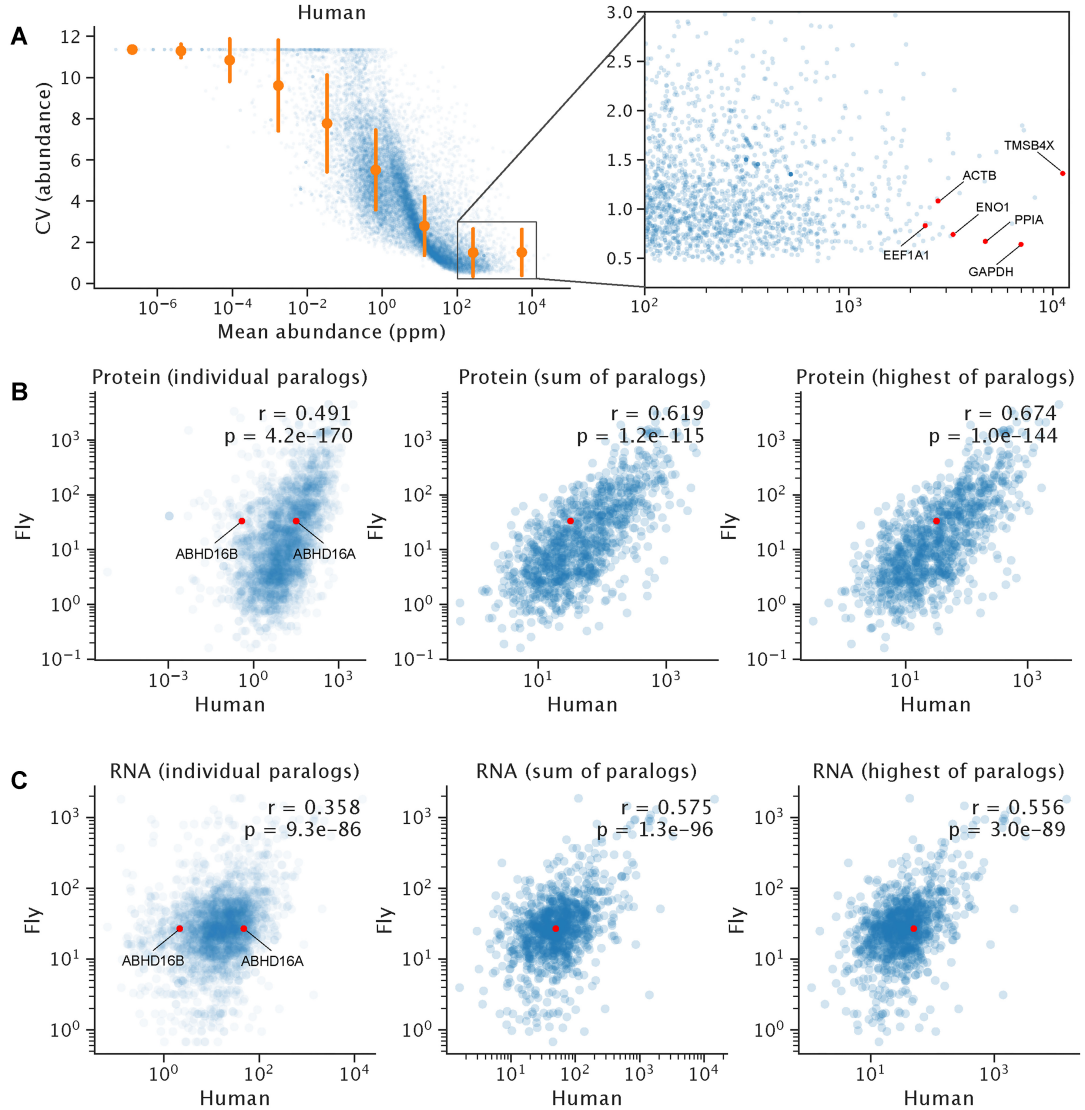
Use cases for PaxDb data. (**A**) CV versus protein abundance for identifying HK proteins. Red dots highlight and annotate known HK genes or stably expressed genes. (**B**) Protein abundance conservation between human and fly homologs. Reduced correlation due to paralog functional divergence is illustrated by increased correlation when considering the sum or the dominant (highest-abundance) human paralog (Left: individual paralogs, Middle: sum of paralogs, Right: dominant paralog). The red dot highlights the example homolog pair: fly (CG1309) and human (ABHD16A/ABHD16B). (**C**) Gene expression conservation between human and fly homologs, showing the same pattern as protein abundance.

Housekeeping (HK) genes typically maintain basic cellular functions and exhibit stable, constitutive expression across different tissues, cell types, and conditions [46]. Because of this universal and stable expression, they are commonly used as reference genes for quantitative polymerase chain reaction normalization [47]. However, regulation occurs at both the RNA and protein levels, and as a result, the set of genes that qualify as HK can differ between RNA and protein. Here, we performed a systematic evaluation of protein abundance and its variability. We used all human datasets of >40% proteome coverage from PaxDb to compute the coefficient of variation (CV) of protein abundance for each gene (Fig. 4A). As expected, CV decreases with increasing abundance. We also compiled the top 50 most abundant genes in five species—human, mouse, chicken, macaque, and pig—along with their CVs (Supplementary Table S1 in Supplementary data). This analysis confirms that commonly used housekeeping genes, such as GAPDH and beta-actin, are also stable at the protein level. In addition, it highlights other highly abundant and low-variation genes across species, including TMSB4X, EEF1A1, PPIA, CFL1, and ENO1. Notably, three of these genes (EEF1A1, CFL1, and ENO1) are embryonic lethal in model organisms, supporting their essential biological roles. [48–50].

#### Dosage conservation and functional divergence of homologs

During evolution, common ancestral genes are passed on to different lineages after speciation events and appear as orthologous genes across species. Independent gene duplication events in each lineage can result in different numbers of paralogs derived from the same ancestral gene. Because homologs share a common origin, their functions are often similar [51]. As redundancy arises, paralogs may evolve independently, leading to loss of function (pseudogenization), partitioning of ancestral functions (subfunctionalization), or the acquisition of new functions (neofunctionalization) [52]. The subfunctionalization model posits that following whole-genome duplication (WGD), paralogs evolve neutrally as long as their combined expression remains approximately constant [53]. Because the major dosage contribution is provided by the highly expressed copy, the lower-expressed paralog is subject to weaker purifying selection and can accumulate functional changes without being purged. Under this model, the summed expression is maintained until the weakly expressed paralogs lose their functional contribution and no longer affect dosage, leaving the dominant paralog as the sole copy under functional and dosage constraint. Such functional shifts in homologs can be detected as a loss of correlation. This has been demonstrated using functional annotation [54] and gene expression data [55]. Here, we extend this approach to the protein level by analyzing PaxDb datasets for human and fly. Because the “2R” WGD events occurred in the human lineage but not in the fly lineage [51], we can investigate the dosage constraints of human paralogs by analyzing correlations in their protein abundance relative to their fly homologs at the bilaterian ancestor level. According to the model, the summed abundance of human paralogs, or the abundance of the dominant paralog, should correlate more strongly with the fly counterpart than the abundances of the individual human paralogs.

To test this hypothesis, we analyzed the subset of orthologs with a one-to-many relationship between fly and human (Fig. 4B). The abundances of the selected fly genes correlated with those of their human homologs (Pearson’s *r* = 0.491). When only the dominant paralog (the one with the highest abundance among the human homologs) was considered, the correlation increased to *r* = 0.674. The summed abundance of paralogs also increased the correlation, albeit to a lesser extent (*r* = 0.619, Steiger’s Z test *P*-value = .0041). This supports the view that, on average, the dominant paralog retains the primary functionality. As an illustrative example, we highlight the fly gene CG1309 and its human homologs ABHD16A and ABHD16B (shown in red). In PaxDb, their mean abundances are 26.9 ppm, 49.4 ppm, and 2.16 ppm, respectively. ABHD16A (558 aa) represents the dominant paralog, retaining the serine hydrolase function of CG1309, whereas ABHD16B (469 aa) has diverged, acquiring hydrolase activity toward glycerophospholipids [56].

In addition, we performed the same analysis using RNA expression data, measured in transcripts per million, from Bgee [57] (Fig. 4C). We selected large-scale single studies from the Bgee collection: SRP012682 (GTEx) for human and SRP045429 (abundance variation) for fly. Compared to protein, the RNA expression correlations of homologs were weaker for individual paralogs (Pearson’s *r* = 0.358). As with protein abundance, correlations increased when using the sum of paralogs (*r* = 0.575) or the dominant paralog (*r* = 0.556). In both cases, protein expression reflected abundance conservation more strongly, with higher cross-species correlations than RNA expression.

Together, these results support neofunctionalization after gene duplication, as evidenced by improved cross-species correlation when considering the dominant paralog or summed abundances.

## Discussion

Reprocessing MS data from scratch offers three main advantages. First, advances in software and algorithms allow more accurate spectrum matching and peptide distinction [13, 28, 29]. Second, updated genome assemblies provide more complete peptide reference libraries. Third, relying on protein identifier-based, previously processed data risks ID loss or mapping errors when identifiers change across database versions (for example at Ensembl). An end-to-end MS data processing workflow ensures direct control over raw data processing, with versioned proteogenomics libraries and versioned software tools, thereby improving data standardization and reproducibility within PaxDb.

The current workflow has several limitations. First, the pipeline uses standard parameters from template workflows and does not automatically adjust to specific experimental setups. Data quality is controlled through PPI-based scoring and minimum required coverage cut-offs: if parameters are misconfigured, quantification results are often abnormally sparse (fewer than 50 peptides identified) and excluded from further steps. For transparency, we release SDRF files recording all parameters for each processed project. Second, peptidase enzymes default to trypsin, but the workflow can recognize common alternatives through pre-loaded FragPipe templates (e.g., LysC, Trypsin+LysC, GluC). While multi-enzyme protocols on the same sample aim to capture more peptides, datasets from different enzymes are not merged and remain as separate entries. Furthermore, enzyme names are not always present in file names, preventing correct parameter parsing and, in some cases, making experiments unprocessable. Third, not all raw data formats are supported. Currently, only DIA experiments are converted to .mzML for DIA-NN; formats such as Agilent Q-TOF 6520 (.d files) are excluded due to the trade-off between adding compatible file formats and increasing pipeline complexity. Fourth, for duplex or triplex labeling (e.g., SILAC and dimethyl labeling [58]), label information (light, medium, heavy) can sometimes be inferred from project descriptions. However, for multiplexed experiments with chemical or isobaric labels (iTRAQ, TMT) where more than three samples are measured in a single file across different channels, an SDRF-type description becomes necessary to process them. Finally, as our filtering depends on publications linked to PX projects, many projects labeled as “Dataset with its publication pending” become unfindable to us when their records are not updated with a PubMed ID following acceptance. In future releases, we plan to run reverse full-text searches to re-identify such projects.

From developing our workflow, we identified two main hurdles to reproducible data reuse. First, many experiments failed to process because files were corrupted or used deprecated formats that could not be opened by the converters. Second, critical information linking data files to experimental conditions was missing in the majority of projects. In our workflow, filename-based grouping recovered nearly 20% of such projects. While FragPipe automatically generates an SDRF file during processing, these files are often not uploaded, or authors may use alternative software without this feature. Widespread adoption of SDRF files is essential for systematic data re-analysis, and we call on proteomics journals to mandate their submission as part of data deposition.

With our unbiased approach to collecting all accessible MS-based proteomics datasets, we are including more non-model organisms. These additions enrich the resource by filling knowledge gaps in sparsely studied branches of the evolutionary tree and can have a disproportionately large impact on understanding evolutionary patterns in protein abundance. However, for species with poorly annotated genomes and less reliable PPI information, quality scores may trend lower even when measurements are accurate and coverage is high. Therefore, quality scores should be interpreted relative to datasets from the same or closely related species.

For the LLM-based curation pre-selection, we use a 50–50 random guess as a baseline performance, which roughly reflects the class imbalance and yields a precision of 0.352. For an improved, but still naive baseline, we then use keywords containing “quant” or “abundance,” mirroring the starting point of manual evaluation, resulting in a precision of 0.512. Any improvement over this already simplifies the selection process. Across all method groups, we observed higher precision and, for some models, similar or improved recall. The classification task remains challenging for two main reasons. First, binary labeling does not capture the underlying reasons for inclusion or rejection defined in our scope. For example, an abstract describing a quantitative proteomics study may emphasize the functional pathway discovered; if labeled positive, the model might incorrectly learn that pathway descriptions alone are indicators of positive labels. Second, the labeled dataset is small relative to the task’s complexity. For certain rejection categories, only a few examples exist from which to learn patterns. As more projects are manually evaluated within the workflow, the resulting larger training set should further improve model performance.

The OpenAI API methods were limited by their inability to learn across examples. Each text was processed with direct instructions but without keeping memory or state between API calls, preventing the model from correcting mistakes as an experienced human evaluator would. Even when the instruction summarized the evaluator’s approach, it could not capture the full thought process behind text labeling. This inherent weakness, combined with the model’s difficulty in retaining cross-sentence logic, explained the moderately improved performance over the baseline.

Conceptually, TM methods are less suited to this classification task, as they summarize the corpus into topic clusters without reference to the training labels. Some topics may align with inclusion or rejection criteria by chance, enabling generalization to unseen texts. The optimization step can produce such models, but performance likely saturates at an F1 score of ∼0.69 due to topic cluster size imbalances and the limits of permutation.

EC methods address the limitations of discrete topic grouping by operating directly on text embeddings. Fine-tuning pre-trained transformer models with labeled data allows them to specialize in the classification task. Because the selected pre-trained models were optimized for different text types (e.g., long inputs, scientific corpus, biomedical corpus), the resulting models brought diverse “understandings” of the text, strengthening the effect of majority voting. This diversity in model specializations likely enhanced the effect of majority voting, producing a precision 6.3 percentage points higher than the TM group despite similar F1 scores for individual models.

It is worth noticing that the method groups show relatively low agreement, suggesting that each method captures different aspects of the text. The keyword method alone achieves 0.512 precision, corresponding to a positive/negative odds ratio of 1.05 (0.512/0.488). When applied to projects already selected by the EC model, keyword filtering yields a near-neutral odds of 1.01 (646/639); when applied to TM-selected projects, the odds drop to 0.85 (624/728), showing slight disagreement. Both are close to the baseline of 1.05 when conditioned on all projects. In contrast, the odds ratios between EC and TM are 0.169 (186/1099) and 0.160 (186/1166), indicating strong disagreement. This likely reflects their conceptual differences: EC models leverage fine-tuned embeddings, while TM clusters topics without label awareness. From a project inclusion perspective, their complementarity is beneficial, as it increases recall. Given the high downstream project drop-out rate, we plan to optimize the classifiers for recall rather than precision.

The current user upload functionality supports only peptide-based quantification results, enabling data quality assessment and reliable comparisons to PaxDb reference data. However, the barrier to reanalyzing proteomics data often lies in the need for substantial computing infrastructure and the complex setup of software environments and input files. For many datasets, processed peptide tables are absent, forcing users to begin from raw data. Uploading gigabytes of raw files is impractical, but future developments could offer on-demand raw data processing, with users supplying the PX project identifier and essential metadata. Such capabilities would further lower the barrier to systematic data reuse and extend PaxDb’s role as an integrative proteomics resource.

## Conclusion and future perspective

With v6.0, PaxDb expands its species and dataset coverage substantially while introducing a reproducible, version-controlled workflow for MS data reprocessing. By combining automated file parsing, standardized quantification pipelines, and quality control based on STRING interaction networks, the database delivers even more consistent and comparable protein abundance estimates across studies. The integration of LLM-based project classification addresses the bottleneck of dataset triage, enabling scalable inclusion of relevant studies from the rapidly growing PX archive. The addition of a user-facing processing interface extends PaxDb’s utility beyond curated references, allowing researchers to assess and benchmark their own measurements. While limitations remain in terms of handling non-standard file formats, complex labeling schemes, and incomplete metadata, these developments set the stage for broader and more systematic integration of proteomics data. As more datasets adopt standardized formats such as SDRF, PaxDb will continue to refine its automated workflows and expand coverage, further supporting quantitative, cross-species protein abundance research.

## Supplementary Material

gkaf1066_Supplemental_Files

## Data Availability

The PaxDb database is freely accessible at https://pax-db.org. Database records from the latest and previous versions are available at https://pax-db.org/downloads. Creative Commons Attribution 4.0 International (CC BY 4.0) applies to all content of PaxDb resource.
