# Supplementary material for: PaxDb v6.0: reprocessed, LLM-selected, curated protein abundance data across organisms
Source: Nucleic Acids Res. 2025 Nov 3;54(D1):D427–39. doi: 10.1093/nar/gkaf1066 (PMC12807614; doi:10.1093/nar/gkaf1066)
Supplement: gkaf1066_Supplemental_Files [file gkaf1066_supplemental_files.zip › Supplementary Figures PaxDb v6.pdf]

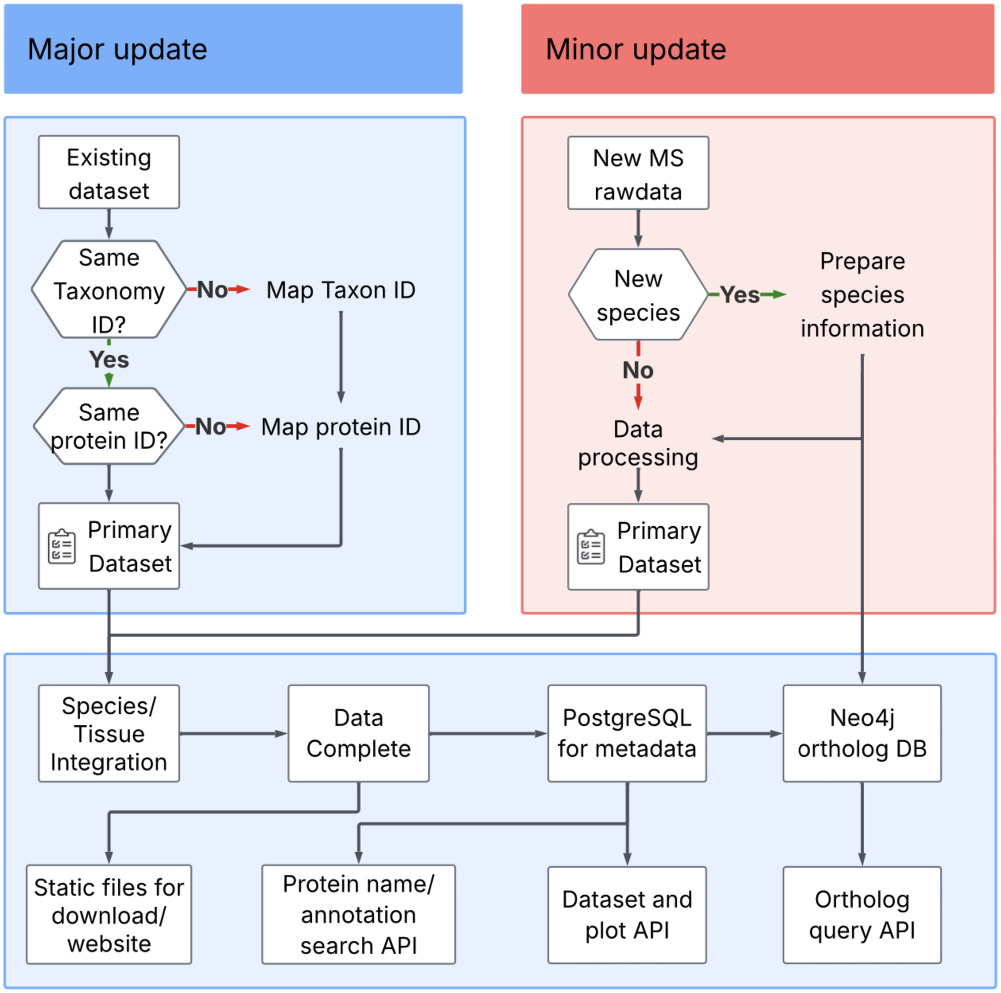

Supplementary Figure 1: Illustration of the version update streamline. Major updates involve mapping existing datasets into new genome versions while minor update involve adding new datasets. The downstream database/downloads building processes are shared by both.

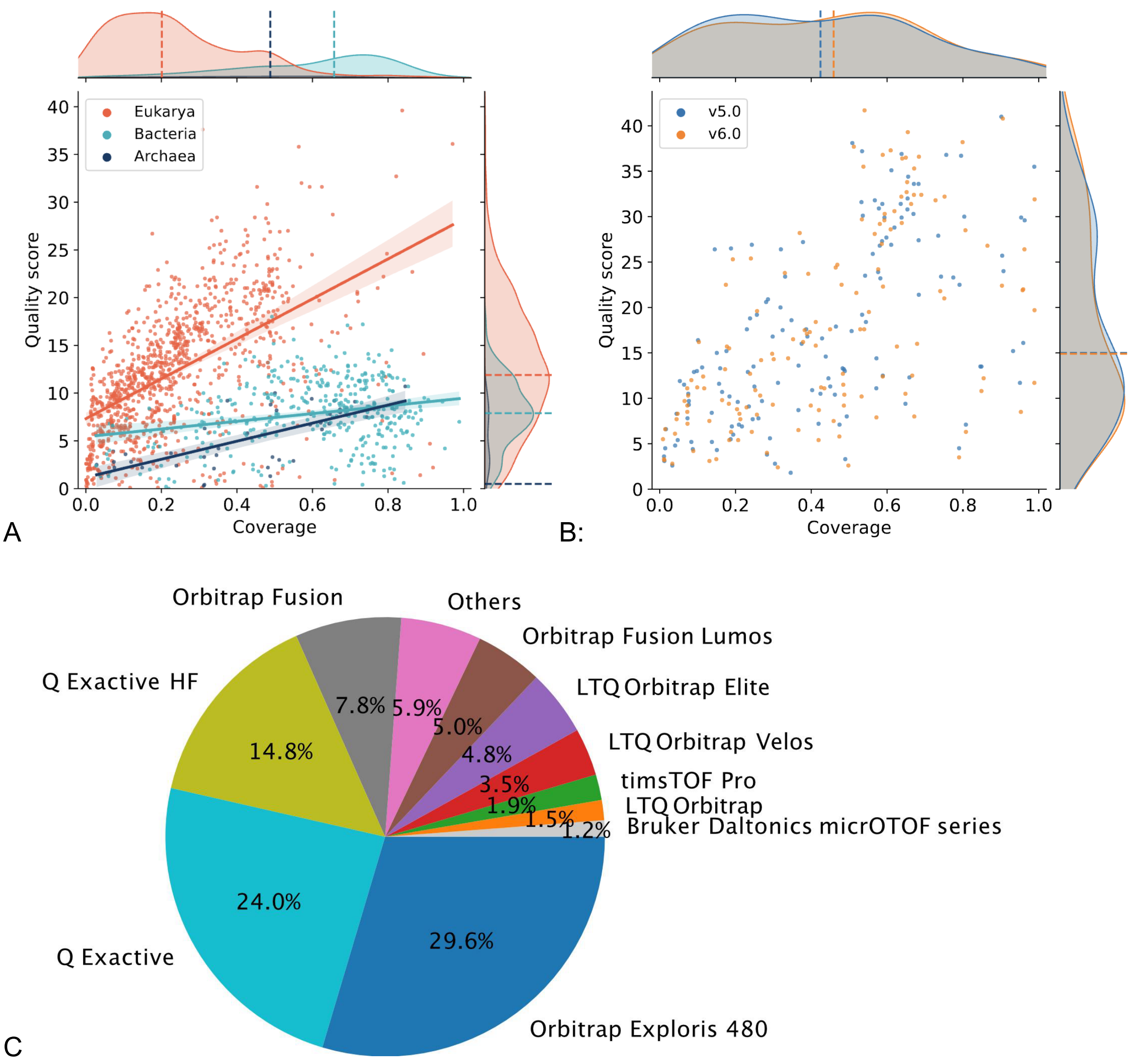

Supplementary Figure 2: Data update statistics. A: The dataset quality score and proteome coverage between Eukarya, Bacteria and Archaea in v6.0 datasets. B: The dataset quality score and proteome coverage on 139 integrated datasets that overlap between v5.0 and v6.0. C: Proportion of mass analyzer instruments for the MS experiments for the datasets.

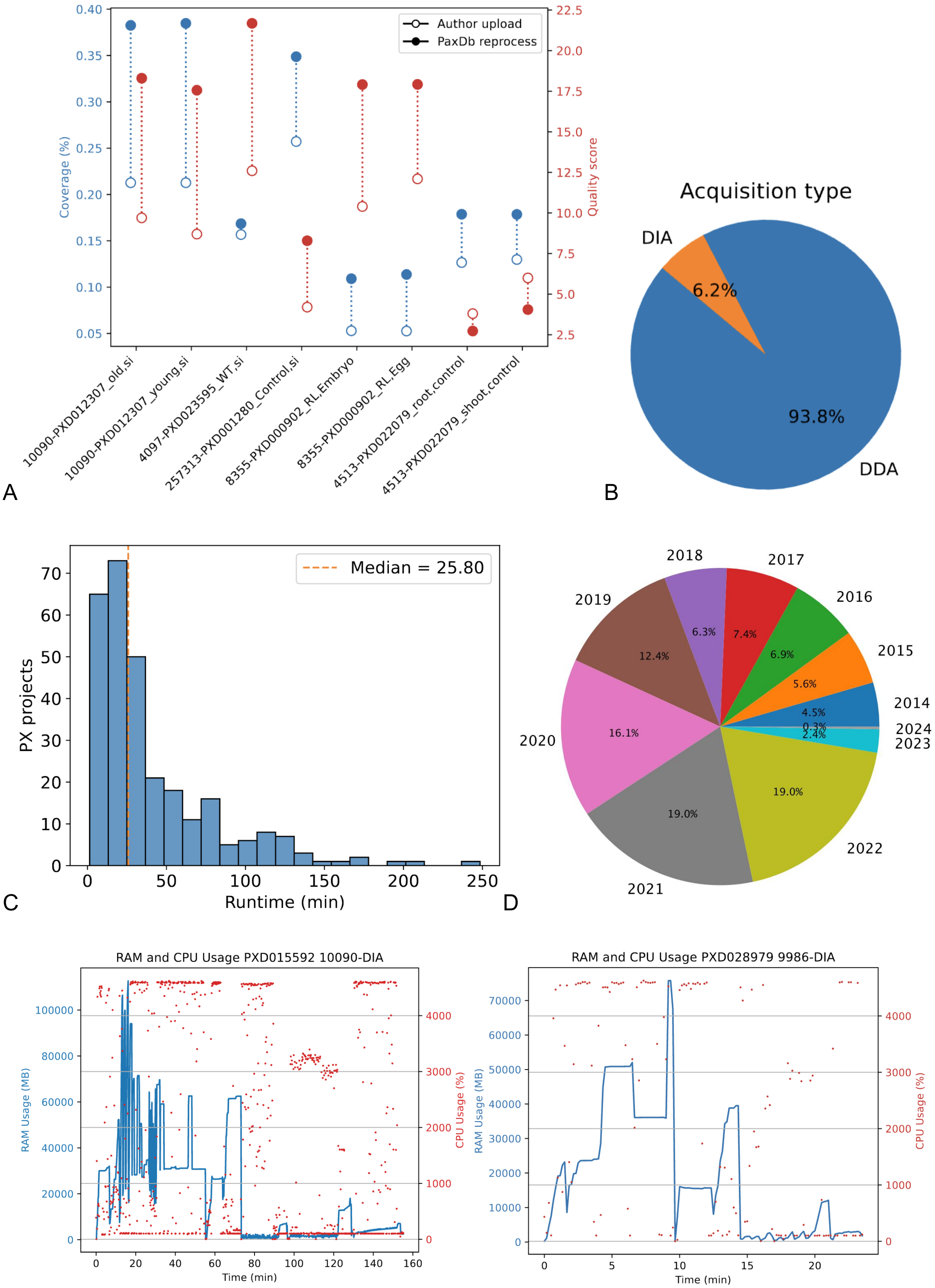

Author upload
PaxDb reprocess
Coverage (%)
Quality score
10090-PXD012307_old.si
10090-PXD012307_young.si
4097-PXD023595_WT.si
257313-PXD001280_Control.si
8355-PXD000902_RL.Embryo
8355-PXD000902_RL.Egg
4513-PXD022079_root.control
4513-PXD022079_shoot.control
A
Acquisition type
DIA
6.2%
93.8%
DDA
B
Median = 25.80
PX projects
Runtime (min)
C
2018
2017
2016
2015
2014
2024
2023
2022
2021
2020
2019
6.3%
7.4%
6.9%
5.6%
4.5%
0.3%
2.4%
19.0%
19.0%
16.1%
12.4%
D
RAM and CPU Usage PXD015592 10090-DIA
RAM Usage (MB)
CPU Usage (%)
Time (min)
RAM and CPU Usage PXD028979 9986-DIA
RAM Usage (MB)
CPU Usage (%)
Time (min)

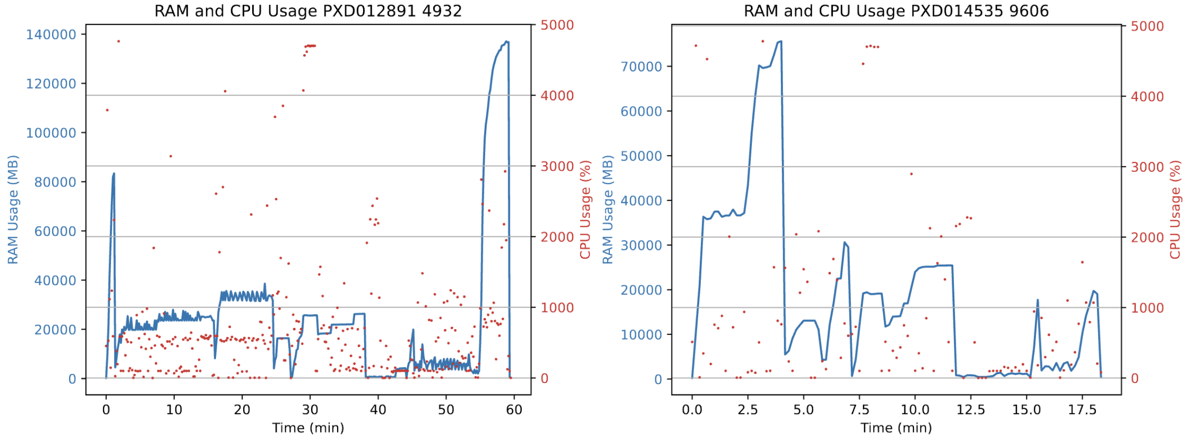

E

F

Peptidase enzyme	No. projects
trypsin (default)	137
gluC	3
LysC	3

Supplementary Figure 3: End-to-end raw data processing pipeline benchmark and summary statistics. A: Comparison of coverage and quality score upon re-analysis on datasets with author-uploaded quantification results. B: The MS experiment acquisition types: DIA and DDA ratio for the added data using the pipeline. C: The runtime profile of the finished PX projects. D: Distribution of publication years for datasets added by the workflow. E: Resource footprint on four example projects. F: Peptidase enzymes from the successfully processed projects.

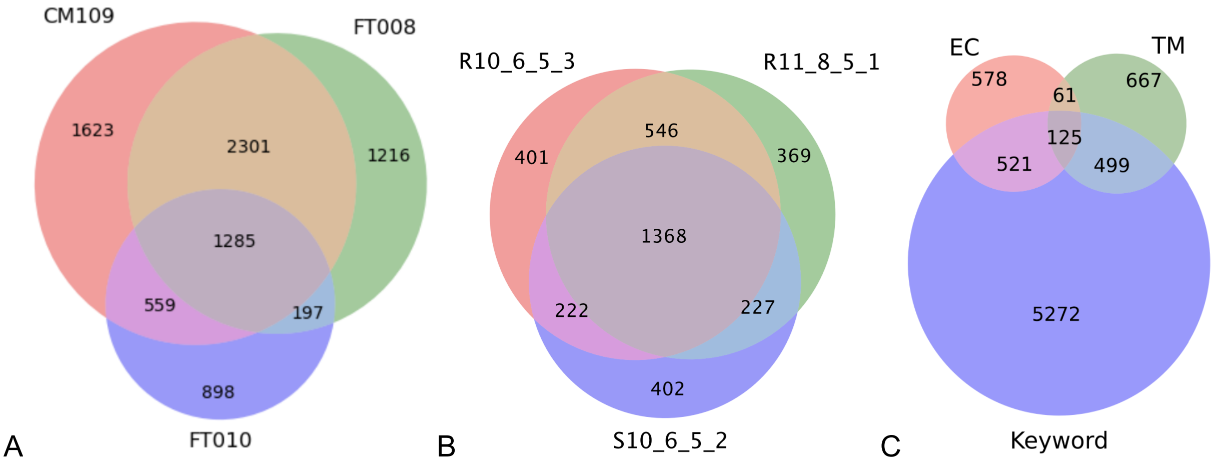

Supplementary Figure 4: LLM model prediction outcome overlap. A: Top 3 embedding classifier (EC) models. B: Top 3 topic modeling (TM)-based models. C: The overlap of consensus projects from EC and TM models with keyword-filtering baseline.

Supplementary Material 1: OpenAI GPT prompt

You are a helpful assistant. I have listed several studies identified by their IDs, along with their titles and abstracts. Please evaluate each study to determine whether it focuses on quantitative proteomics Below are specific criteria for inclusion and exclusion:

Include a study (mark as 'yes') if it:

1 Mentions differential expression or changed expression of proteins.
2 Involves quantitation of a large number of proteins in the proteome.
3 Quantifies the whole proteome for entire organisms or tissues in higher organisms
4 Quantifies proteomes under different conditions with a control sample.

Exclude a study (mark as 'no') if it:

1 Only identifies proteins instead of quantifying them.
2 Focuses on the quantitation of proteins within a specific functional pathway rather than the entire proteome.
3 Studies environmental samples consisting of mixed organisms.
4 Studies disease or treatment samples without healthy controls.
5 Studies sub-cellular organelles exclusively.
6 Studies extracellular components like secretion or body fluids exclusively.
7 Quantifies viral proteins exclusively.
8 Quantifies fractions of the proteome separated by fractionation techniques.
9 Is primarily about developing software or protocols.
