# Supplementary figures and images for: PaxDb v6.0: reprocessed, LLM-selected, curated protein abundance data across organisms
Source: Nucleic Acids Res. 2025 Nov 3;54(D1):D427–39. doi: 10.1093/nar/gkaf1066 (PMC12807614; doi:10.1093/nar/gkaf1066)

Tissue-specific data count overview

Species | Organ

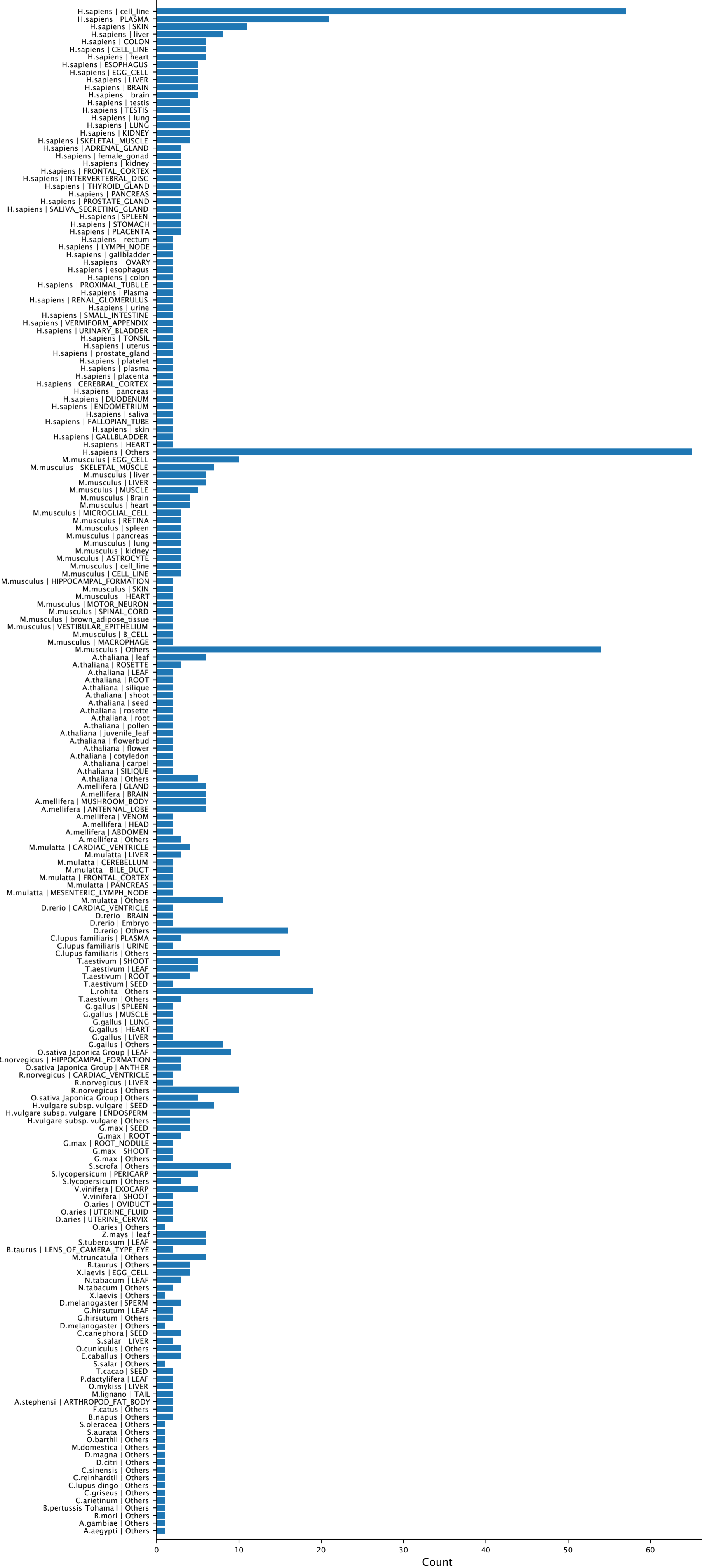

Supplement: gkaf1066_Supplemental_Files [file gkaf1066_supplemental_files.zip › species_organ_bar.pdf]
